# La place de la thoracoscopie dans la prise en charge des pathologies thoraciques: à propos de 104 cas

**DOI:** 10.11604/pamj.2015.21.42.6041

**Published:** 2015-05-21

**Authors:** Marwane Lakranbi, Sani Rabiou, Jamal Ghalimi, Ibrahim Issoufou, Yassine Ouadnouni, Mohamed Smahi

**Affiliations:** 1 Service de Chirurgie Thoracique, CHU Hassan II, Fès, Maroc; 2 Université Sidi Mohamed Ben Allah, Faculté de Médecine et de Pharmacie, Fès, Maroc

**Keywords:** Biopsie, métastases, thoracoscopie, Biopsy, metastases, thoracoscopy

## Abstract

**Introduction:**

La thoracoscopie est l'exploration endoscopique de la cavité pleurale, des organes avoisinants (diaphragme, péricarde, médiastin) et du poumon. Le but de ce travail se veut d'abord didactique; décrivant la thoracoscopie, ses techniques ainsi que sa place dans la prise en charge de la pathologie thoracique (indications et perspectives thérapeutiques) et informatif en rapportant l'expérience de notre équipe.

**Méthodes:**

Il s'agit d'une étude rétrospective intéressant 104 thoracoscopies à visée diagnostique et/ou thérapeutique réalisées au service de chirurgie thoracique du Centre Hospitalier Universitaire Hassan II de Fès, sur une période de 04 ans (Août 2008-décembre 2012). Nous avons exclu de notre étude les cas ayant bénéficié d'une médiastinoscopie ainsi que les cas ayant bénéficié d'une thoracoscopie dans le cadre des traumatismes fermés du thorax ou des plaies thoraciques.

**Résultats:**

L’ âge moyen des patients est de 47 ans, avec des extrêmes allant de 18 à 80 ans, et une légère prédominance masculine à 54%. La thoracoscopie est d'ordre pleural chez 86 patients, pulmonaire chez 10 patients et médiastinale chez 8 patients. La thoracoscopie avait une indication à visée diagnostique chez 87 cas et thérapeutique chez 52 patients (talcage dans 45 cas, décortication pleuropulmonaire dans 2 cas, résection de kystepleuro-péricardique dans 2 cas, cure de pneumothorax dans 2 cas et une fenêtre péricardique). L’évolution post opératoire etait marquée par une amélioration clinico-radiologique chez 40 malades, 11 ont présenté une amélioration clinique seule, 6 ont présenté une persistance ou une récidive de l’épanchement.

**Conclusion:**

La thoracoscopie représente un réel gain en matière de diagnostic de certaines pathologies intra-thoraciques. Son intérêt thérapeutique limité doit être éventuellement étendu grâce à la chirurgie thoracique vidéo assistée, qui est une technique récente fiable avec une limitation de la durée d'hospitalisation et de la morbidité.

## Introduction

La thoracoscopie est l'exploration endoscopique de la cavité pleurale, des organes avoisinants et du poumon. C'est une technique à visée diagnostique et/ou thérapeutique [1]. Elle a été mise au point pour la première fois par JACOBEUS en 1905 [2], mais c'est au cours de ces dernières années que la thoracoscopie a pris une ampleur importante. La thoracoscopie peut être réalisée à visée diagnostique et/ou thérapeutique dans différentes affections pleurales (pleurésies à liquide clair, purulent ou hémorragique..), pulmonaires (maladies interstitielles pulmonaires, nodules pulmonaires uniques ou multiples, pneumothorax, bulles d'emphysème..), médiastinales (exérèses tumorales..), cardiaques (péricardite liquidienne..), et neurochirurgicales (les sympathectomies thoraciques, les vagotomies tronculaires, la chirurgie du rachis thoracique.) [1]. Notre travail se veut d'abord didactique; décrivant la thoracoscopie, ses techniques ainsi que sa place dans la prise en charge de la pathologie thoracique (indications et perspectives thérapeutiques) et informatif en rapportant l'expérience de notre équipe dans ce domaine.

## Méthodes

Il s'agit d'une étude rétrospective intéressant 104 thoracoscopies réalisées au service de chirurgie thoracique du Centre Hospitalier Universitaire Hassan II de Fès, sur une période de 04 ans (Août 2008-décembre 2012). Nous avons inclus dans cette étude tous les patients ayant nécessité un actethoracoscopique à visée diagnostique et/ou thérapeutique, à tout âge et ayant une pathologie pleurale, pulmonaire ou médiastinale pour lesquelles une exploration thoracoscopique s'est avérée nécessaire afin d’établir un éventuel diagnostique ou de réaliser une cure chirurgicale. Nous avons exclu les cas ayant bénéficié d'une médiastinoscopie ainsi que les cas ayant bénéficié d'une thoracoscopie dans le cadre des traumatismes fermés du thorax ou des plaies thoraciques. Les données suivantes, ont été consignées chez tous les patients inclus dans l’étude: les données anamnestiques; les données de l'examen clinique; les données des examens d'imagerie; la technique de l'intervention chirurgicale; les données de l'exploration; les résultats de l’étude anatomopathologique; l’évolution l'analyse statistique a été réalisée grâce au logiciel Epi Info.

## Résultats

**Figure 1:**
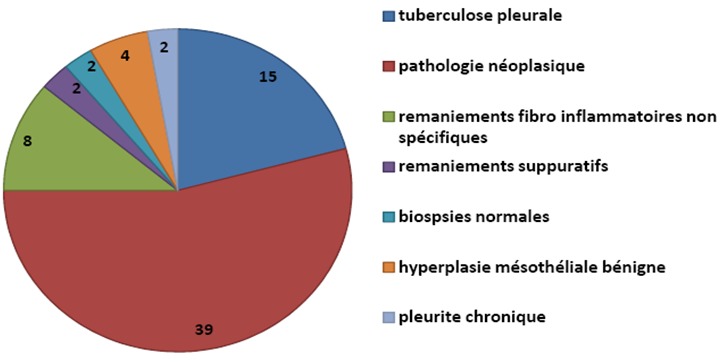
Les différentes indications pleurales à visée diagnostique

**Figure 2:**
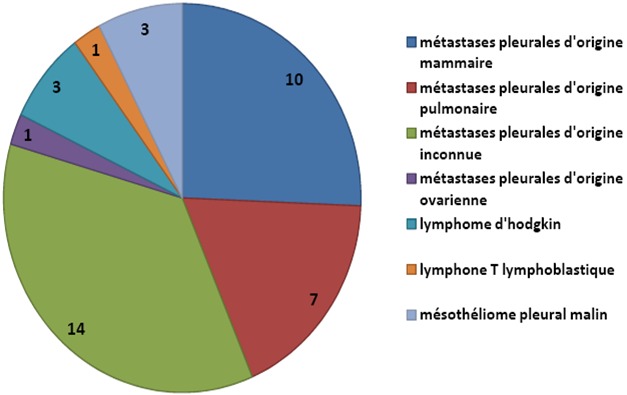
Les différents diagnostics histologiques des localisations pleurales néoplasiques

**Figure 3:**
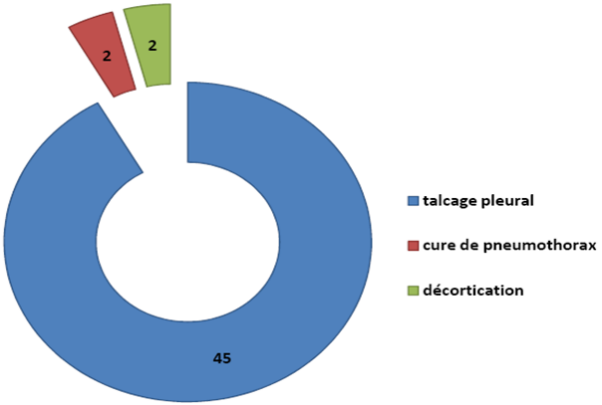
Répartition des patients selon le geste chirurgical pleural realize

L’âge moyen des patients est de 47 ans, avec des extrêmes allant de 18 à 80 ans, et une légère prédominance masculine à 54%. 28 patients sont des tabagiques chroniques, 19 patients sont suivis pour une pathologie néoplasique et seulement 4 patients ont des antécédents d´une tuberculose thoracique. Un syndrome d’épanchement liquidien était présent chez 83 patients, dont 56 patients ont bénéficié auparavant d´une ponction biopsie pleurale (au moins deux tentatives) revenue non concluantes. Sur les 104 thoracoscopies, 102 ont été réalisées sous anesthésie générale et 2 sous sédation compte tenu de l’état clinique du patient incompatible avec un décubitus dorsal. L'intubation était sélective pour 94 patients et normale pour 10 patients. Nous avons reparties les indications de la thoracoscopie non seulement selon les pathologies pleurale, pulmonaires et médiastinales, mais aussi selon qu'elle soit réalisée dans le cadre diagnostic et ou thérapeutique:

**Indications pleurales**: Les indications pleurales diagnostiques concernent 72 patients et les résultats sont résumés dans la Figure 1. Chez 39 patients qui ont présentés une pathologie néoplasique, les diagnostics histologiques sont repartis sur la Figure 2. La Figure 3 nous donne la répartition des patients chez qui la thoracoscopie a été réalisée dans un but thérapeutique. Elle nous a permis de faire un talcage pleural, une cure du pneumothorax et encore une décortication.

**Figure 4:**
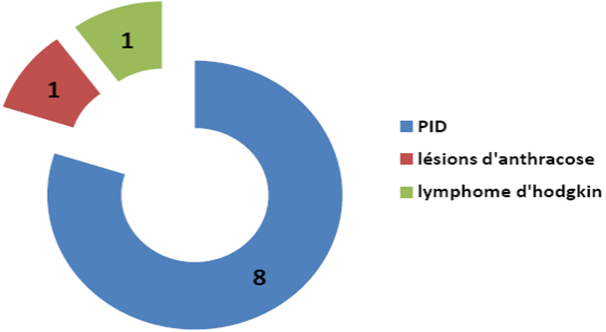
Répartition des Résultats histologiques des différentes biopsies pulmonaires par thoracoscopie

**Indications pulmonaires**: Concernant les pathologies pulmonaires, nous avons réalisés 10 biopsies pulmonaires par thoracoscopie exclusive dont les résultats histologiques sont représentés sur la Figure 4.

**Figure 5:**
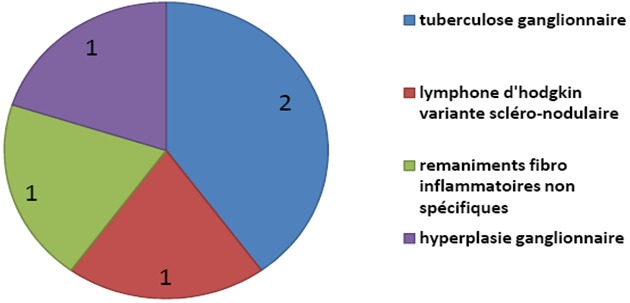
Les résultats histologiques des différentes biopsies médiastinales par thoracoscopie

**Indications médiastinales**: La thoracoscopie pour les pathologies médiastinales a été réalisée dans un but diagnostic chez 5 patients. Il s'agissait de 3 biopsies des ganglions médiastinaux dont 2 au niveau sous carénaires et 1 au niveau de la fenêtre aorto-pulmonaire. Pour 1 patient la biopsie a été réalisée au niveau d'une masse médiastinale et pour un autre patient une biopsie concomitante d'une masse médiastinale ainsi que des adénopathies était nécessaire. Les résultats histopathologiques des biopsies de la pathologie médiastinale sont présentés dans la Figure 5. Les autres pathologies médiastinales étaient représentées par 2 kystes pleuropéricardiques pour lesquelles nous avons réalisé une exérèse totale par thoracoscopie et une 3ème patiente suivie pour cancer du sein et qui a présenté une pleuropéricardite carcinomateuse pour laquelle une fenêtre pleuropéricardique était nécessaire.

**L’évolution des patients**: L’évolution post opératoire étaitmarquée par une amélioration clinico-radiologique chez 40 malades, 11 ont présenté une amélioration clinique seule, 6 ont présenté une persistance ou une récidive de l’épanchement. Par ailleurs nous avons noté un seul cas de décès survenu en postopératoire immédiat chez un patient qui a présenté un œdème aigu du poumon d´origine cardiogénique.

## Discussion

**Figure 6:**
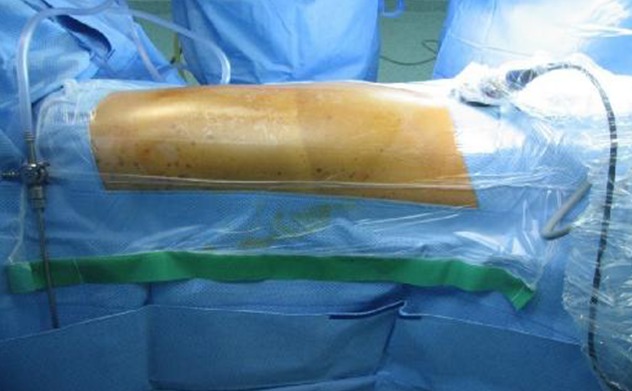
Installation du patient en position opératoire (Archive du Service de chirurgie thoracique, CHU Hassan II, FES)

**Figure 7:**
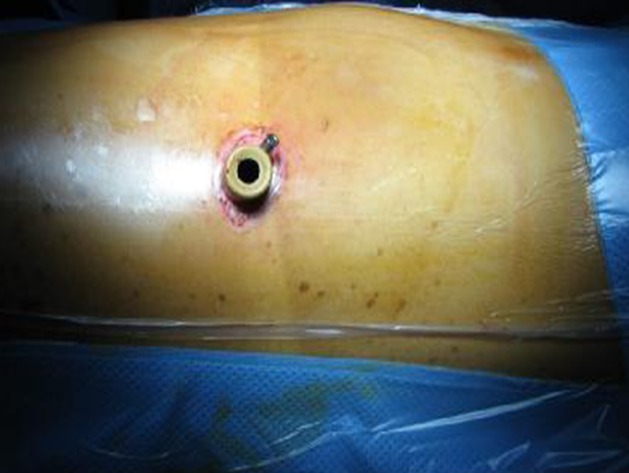
Position du trocart au niveau du 7ème espace intercostal (Archive du service de chirurgie thoracique, CHU Hassan II, FES)

La thoracoscopie est une technique chirurgicale qui se pratique souvent sous anesthésie générale avec une intubation trachéale le plus souvent sélective. Chez les patients à haut risque ou en mauvais état général, ou en cas d'insuffisance cardio-respiratoire contre-indiquant une anesthésie générale, une sédation ou une anesthésie locale est privilégiée [3]. L´examen se fait à jeûne en position couchée, sur le côté opposé à celui à investiguer (Figure 6) ce qui donne au chirurgien une très bonne exposition du hile pulmonaire [4]. La réalisation d'un geste par thoracoscopie nécessite un équipement dédié à cet effet [5]. La technique de la thoracoscopie médicale [6] utilise un thoracoscope avec une source de lumière froide que l'on introduit dans le thorax à l'aide d'un premier trocart (Figure 7). Le plus souvent on est amenés à introduire un deuxième trocart qui nous permettra non seulement de réaliser une biopsie pleurale, mais aussi de réaliser une symphyse pleurale en injectant du talc [7] préalablement préparé. En ce qui concerne la vidéo thoracoscopie chirurgicale [5, 7], elle se pratique sous anesthésie générale et nécessite une intubation sélective de manière à exclure le poumon permettant aux chirurgiens de travailler en toute liberté dans une cavité pleurale libre. Elle repose sur le principe de trois trocarts en triangulation, le 3ème trocart inférieur étant utilisé pour l'optique et le trocart latéral utilisé pour introduire les instruments endoscopiques spécifiques. Cette technique permet de réaliser des gestes surtout thérapeutiques à type d'exérèse parenchymateuse atypique « wedge résection » pour nodule pulmonaire ou d'exérèse de tumeurs médiastinales dont la taille n'excède généralement pas 5 cm ou bien à visée diagnostique à type de biopsie d'une adénopathie inaccessible à la médiastinoscopie concernant les sites ganglionnaires médiatisnaux N° 5, 6, 7 et 9. Comme dans la chirurgie conventionnelle, un ou deux drains sont mis en place de façon systématique à la fin du geste, afin de drainer l’épanchement résiduel. Une thoracoscopie chirurgicale est dite vidéo-assistée ou VATS [8–10], si elle répond à une certaine exigence définissant la technique dite à thorax fermé décrite par N. Shigemura: pas d’écarteur; si une incision d'appoint est faite, elle n'est pas utilisée pour opérer; toute l'intervention est faite par thoracoscopie; seuls des instruments endoscopiques sont utilisés; les instruments endoscopiques sont introduits par l'intermédiaire d'une minithoracotomie laissant seulement une petite cicatrice [11]. Dans notre série, une conversion en thoracotomie était nécessaire chez 8 patients soit 7,7%. Les causes des conversions étaient: une désaturation à moins de 30% chez un patient suite à une intubation sélective, un cas de lésion de l'artère mammaire interne ayant nécessité une conversion pour assurer une hémostase satisfaisante, pour les autres cas il s'agissait de la présence d'adhérences gênant le geste opératoire.

Les biopsies étaient non contributives chez 8 patients parmi 72 soit 11,1%, toutefois; aucun redressement diagnostic n´a été rapporté chez ces malades ultérieurement. Fletcher dans sa série rapporte 8% des cas des biopsies pleurales non concluantes [12]. Une patiente avait bénéficié de 3 thoracoscopies à 1 an d'intervalle avant de poser le diagnostic d'un « mésothéliome pleural basaloide ». Le mésothéliome pleural malin était noté dans notre série dans 4,1% des cas seulement, alors qu'il présentait 62% des cas dans la série de Fletcher, 40% des cas dans la série Hadjer [13] et 11% des cas dans la série Garrouch [14]. La tuberculose était notée dans 20,8% des cas dans notre série, dans 22% des cas dans la série Jabri [15], dans 78,5% des cas dans la série El Kard et dans 20% des cas dans la série Hadjer. Ce taux élevé constaté dans la série El kard s'expliquerait par le type de recrutement des patients dont 98% avaient une altération de l’état général chez qui la thoracoscopie a été indiquée devant une pleurésie à liquide claire sans épuisé toute les possibilités diagnostiques. Le cancer primitif n'a pu être étiqueté malgré l’étude histologique dans 19,4% des cas dans notre série, dans 12% des cas dans la série El Kard [16], dans 10% des cas dans la série Hadjer et dans 9% des cas dans la série Garrouch. L'origine néoplasique a été précisée d'une manière générale dans 49% des cas dans la série de Jabri, dans 7,4% des cas dans la série El kard, dans 19% des cas dans la série Hadjer et dans 54,16% des cas dans notre série.4 patients se sont compliqués d'un empyème pleural (4,6%) dans notre série, alors que les complications étaient dominées par les douleurs thoraciques (65%), la fièvre (50%) et l'empyème (2%) dans la série Garrouch. La place de la thoracoscopie dans l'exploration du médiastin reste limitée, dans le sens que d'autres moyens sont largement indiqués, permettant d’éviter pour le patient une intubation sélective, une greffe pleurale par le processus néoplasique. Toutefois; la thoracoscopie nous permet de réaliser des gestes combinés à savoir: biopsie pleurale, talcage pleural en plus de la biopsie de la masse ou du ganglion médiastinal ou bien de la fenêtre aorto-pulmonaire non accessibles à la médiastinoscopie cervicale. Enfin au niveau du poumon, notre expérience est limitée aux biopsies pulmonaires réalisées par thoracoscopie et concerne essentielement des patients suivis en pneumologie pour une pneumopathie interstitielle diffuse chronique.

## Conclusion

La thoracoscopie représente un réel gain en matière de diagnostic de certaines pathologies intra-thoraciques. Son intérêt thérapeutique limité doit être éventuellement étendu grâce à la chirurgie thoracique vidéo assistée, qui est une technique récente fiable avec une limitation de la durée d'hospitalisation et de la morbidité. Actuellement, grâce à l'introduction d'une instrumentation chirurgicale plus sophistiquée, cette technique est devenue de plus en plus pratiquée par les chirurgiens thoraciques dans un but essentiellement thérapeutique permettant la réalisation d'exérèses plus ou moins importantes.
